# Mitochondrial RNA–Type I Interferon Axis in Sjögren’s Disease: Molecular Mechanisms and Translational Implications

**DOI:** 10.3390/ijms27167295

**Published:** 2026-08-15

**Authors:** You-Jung Ha, Yeon Bi Han, Keun-Suh Kim, Woo-Jin Jeong, Joon Young Hyon, Yoosik Kim, Yun Jong Lee

**Affiliations:** 1 Division of Rheumatology, Department of Internal Medicine, Seoul National University Bundang Hospital, Seongnam 13620, Republic of Korea; hayouya@snubh.org; 2 Department of Internal Medicine, Seoul National University College of Medicine, Seoul 03080, Republic of Korea; 3 Department of Pathology and Translational Medicine, Seoul National University Bundang Hospital, Seongnam 13620, Republic of Korea; yeonbihan@snubh.org; 4 Department of Periodontology, Section of Dentistry, Seoul National University Bundang Hospital, Seongnam 13620, Republic of Korea; alienhd@snubh.org; 5 Department of Otorhinolaryngology-Head & Neck Surgery, Seoul National University Bundang Hospital, Seongnam 13620, Republic of Korea; safar@snubh.org; 6 Department of Ophthalmology, Seoul National University Bundang Hospital, Seongnam 13620, Republic of Korea; jyhyon@snubh.org; 7 Center for RNA Research, Institute for Basic Science, Seoul 08826, Republic of Korea; ysyoosik@kaist.ac.kr; 8 Department of Chemical and Biomolecular Engineering, Korea Advanced Institute of Science and Technology, Daejeon 34141, Republic of Korea; 9 Department of Medical Device Development, Seoul National University College of Medicine, Seoul 03080, Republic of Korea

**Keywords:** Sjögren’s disease, interferon signature, mitochondrial double-stranded RNA, salivary gland epithelial cells, innate immunity, biomarkers

## Abstract

Sjögren’s disease (SjD) is a systemic autoimmune disease characterized by exocrine dysfunction, lymphocytic infiltration of the exocrine glands, and prominent activation of the interferon (IFN) pathway. Although IFN signatures are recognized as a central feature of SjD, endogenous triggers that sustain chronic IFN-driven inflammation remain incompletely understood. Mitochondrial RNAs (mtRNAs), particularly double-stranded species, have recently emerged as immunostimulatory molecules capable of linking mitochondrial stress to innate immune activation. In this review, we discuss the biological basis of mtRNA biogenesis and processing, the formation and mislocalization of mitochondrial double-stranded RNAs, and their recognition by innate immune sensors relevant to induction of type I IFN. We also describe how epithelial stress, mitochondrial dysfunction, and mtRNA accumulation amplify IFN-rich inflammatory circuits and glandular injury in SjD. Experimental studies in salivary gland epithelial models and SjD-relevant tissues support a mechanistic role for the mtRNA–type I IFN axis, while emerging clinical data suggest that extracellular mtRNA levels in saliva and plasma may have potential as biomarkers of disease activity and patient stratification. Although current evidence remains limited, the mtRNA–type I IFN axis provides a biologically plausible link between epithelial stress and immune dysregulation, and may have translational implications in SjD.

## 1. Introduction

Sjögren’s disease (SjD; formerly Sjögren’s syndrome) is a chronic systemic autoimmune disease characterized by lymphocytic infiltration of the exocrine glands, epithelial dysfunction, anti-Ro antibody presence, and various extra-glandular manifestations. Its pathogenesis remains incompletely understood, although it is considered multifactorial, involving genetic and environmental susceptibility and dysregulation of innate and adaptive immunity. Among the immunologic abnormalities described in SjD, activation of the interferon (IFN) pathway is regarded as a key pathogenic characteristic [1]. Type I IFN links innate immune sensing to sustained epithelial and adaptive immune activation. Increased expression of IFN-stimulated genes (ISGs) in target tissues and peripheral blood has been consistently reported. However, the upstream endogenous ligands that maintain chronic non-infectious IFN activation in SjD remain incompletely understood.

Recent studies have provided evidence that mitochondrial stress and the release of mitochondria-derived immunoregulatory molecules, particularly nucleic acids with features recognized by antiviral pattern-recognition receptors, can contribute to innate immune activation [2]. Under conditions of cellular stress, defective RNA quality control, or mitochondrial membrane permeabilization, mitochondrial nucleic acids, especially double-stranded species, can engage cytosolic and endosomal nucleic acid-sensing pathways and promote IFN-related responses. In SjD, this concept is of particular interest because salivary gland epithelial cells (SGECs) are regarded as active participants in the initiation and perpetuation of disease rather than as passive targets of professional immune cells. Other classes of RNAs, involved in non-infectious type I IFN activation, include LINE-1-derived transcripts, Alu-associated RNAs, and endogenous retroviral RNAs (Table 1). However, disease-specific evidence supporting their role in SjD remains limited or has largely been extrapolated from broader autoimmune contexts, whereas direct mechanistic and clinical data for mitochondrial RNAs (mtRNAs) in SjD are increasingly available.

Consequently, this review focuses on current knowledge on the interplay between mtRNAs and type I IFN signaling in SjD. It discusses the biological bases of mtRNA biogenesis and surveillance, the formation and mislocalization of mitochondrial double-stranded RNAs (mt-dsRNAs), the sensors and regulatory proteins relevant to this mtRNA-type I IFN axis, evidence linking mtRNA dysregulation and SGEC dysfunction, and the translational potential of mtRNA-based biomarkers for disease monitoring and patient stratification in SjD. Compared with the other candidate endogenous RNA pathways summarized in Table 1, mtRNA dysregulation is supported by a relatively integrated body of mechanistic and emerging clinical evidence in SjD. We therefore propose that mtRNA dysregulation represents a disease-relevant mechanistic bridge linking epithelial mitochondrial stress to sustained IFN activation and may constitute a potentially actionable component of SjD pathobiology.

## 2. Literature Search Strategy

The literature was identified through PubMed and Google Scholar without restriction on publication date, covering articles available through 20 July 2026. Search terms included combinations of “Sjögren’s syndrome,” “Sjögren’s disease,” “mitochondrial RNA,” “mtRNA,” “mitochondrial double-stranded RNA,” “mt-dsRNA,” “type I interferon,” “interferon signature,” “MDA5,” “RIG-I,” “MAVS,” “POLRMT,” “RNA degradation,” “RNA modification,” “mitochondrial dysfunction,” and “mitophagy.” Reference lists of relevant original articles and reviews were also screened to identify additional studies. Priority was given to studies using samples from patients with SjD or SjD-specific experimental models. When direct SjD evidence was unavailable, mechanistically relevant studies from other autoimmune diseases or experimental contexts were included and identified as such. Because this is a narrative review, formal systematic study selection and risk-of-bias assessment were not performed.

## 3. Interferon Signature in SjD

### 3.1. Interferon Pathways in the Immunopathogenesis of SjD

IFNs are key cytokines at the interface of innate and adaptive immunity. Based on phylogenetic lineage, IFNs are classified into four groups; however, as type IV IFNs are absent in mammals, the mammalian immune response is mediated by type I (IFN-α, β, ε, κ, ω), type II (IFN-γ), and type III (IFN-λ1–4) [2,10,11]. IFN pathways occupy a central position in the immunopathogenesis of SjD because they integrate distinct yet overlapping immune programs, including nucleic acid-driven type I and III IFN responses and immune-cell-derived type II IFN signaling, thus linking early innate immune dysregulation with chronic autoimmune inflammation [12,13,14].

Type I IFNs signal primarily through Janus kinase 1 (JAK1) and tyrosine kinase 2 (TYK2), leading to signal transducer and activator of transcription (STAT) 1 and STAT2 phosphorylation. The phosphorylated STAT1–STAT2 heterodimer associates with interferon regulatory factor 9 (IRF9) to form the interferon-stimulated gene factor 3 (ISGF3) complex, which binds to IFN-stimulated response elements (ISREs). They may also induce γ-activated sequence (GAS)-dependent transcription via STAT1 homodimers. Type III IFNs follow a broadly similar downstream signaling cascade through the interferon lambda receptor 1 (IFNLR1)/interleukin-10 receptor subunit beta (IL10RB) receptor complex, although their biological effects are largely restricted to epithelial/barrier tissues. In contrast, type II IFN signals through the interferon gamma receptor 1 (IFNGR1)/interferon gamma receptor 2 (IFNGR2) receptor complex via JAK1 and JAK2, leading predominantly to the formation of STAT1 homodimers that bind GAS elements and modulate immune responses.

All three classes of IFN can contribute to SjD in a compartment- and cell type-dependent manner. These cytokines are produced by various cells in response to microbial or self-derived nucleic acids (Table 1) through pattern-recognition receptors (PRRs). In the autoimmune setting, endogenous nucleic acids released from stressed or injured cells engage endosomal and cytosolic PRRs, including Toll-like receptors (TLRs), retinoic acid-inducible gene I (RIG-I)-like receptors (RLRs), such as RIG-I and melanoma differentiation-associated gene 5 (MDA5), and cyclic GMP-AMP synthase (cGAS)–stimulator of interferon genes (STING)-related pathways [8]. Through downstream JAK/STAT signaling, these pathways amplify antigen presentation, chemokine production, and inflammatory cytokine networks, ultimately promoting dendritic cell (DC), T-cell, and B-cell activation [2].

In this regard, IFN signaling is not just a secondary consequence of inflammation, but a major molecular framework through which innate sensing of danger signals is translated into persistent autoimmune responses.

### 3.2. Type I, Type II, and Type III IFN Signatures in SjD

**Figure 1:**
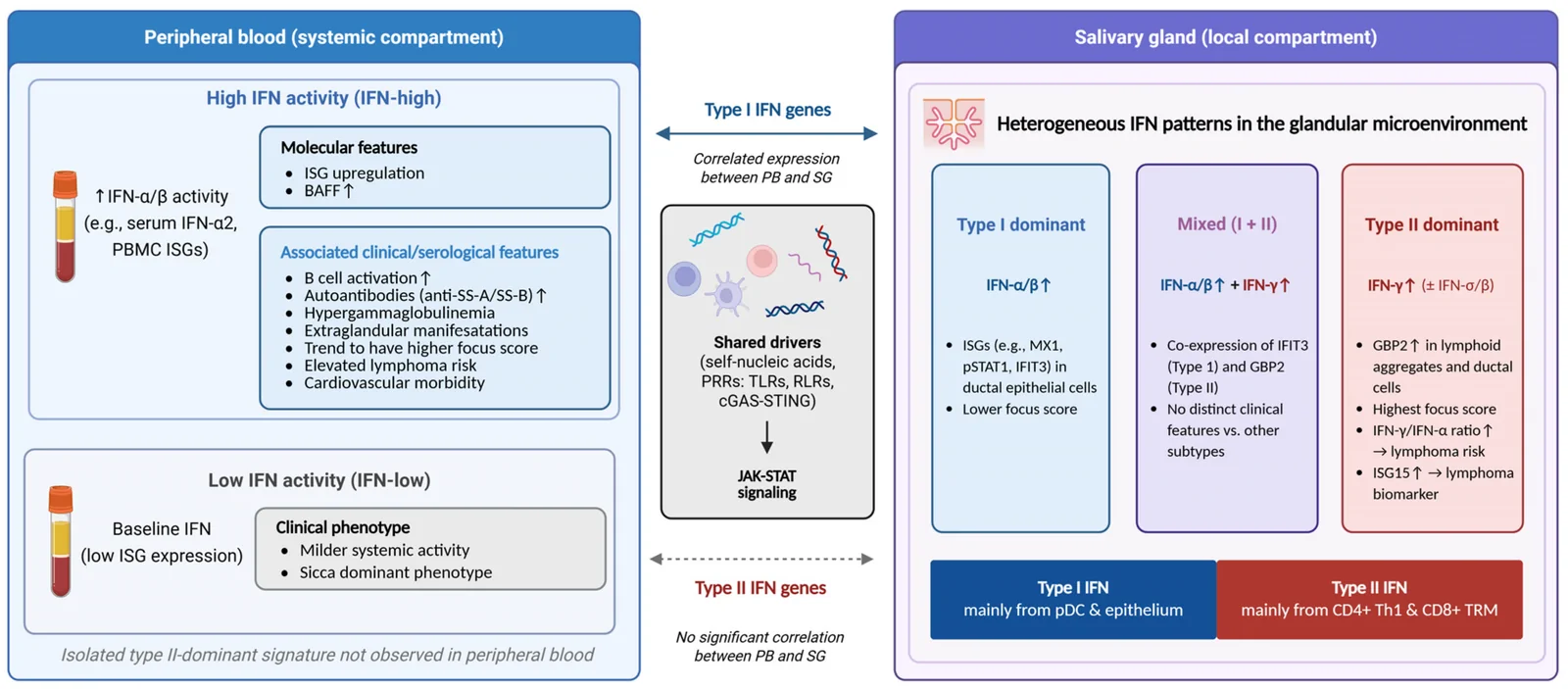
Compartmentalized and heterogeneous IFN landscape in SjD. Type I IFN activity predominates in peripheral blood, where IFN-high status is associated with ISG upregulation, BAFF expression, and systemic autoimmune features, whereas IFN-low patients exhibit a sicca-dominant phenotype. The salivary gland microenvironment displays heterogeneous IFN patterns (type I dominant, mixed, and type II dominant); type II IFN enrichment is linked to higher focus scores and elevated risk of lymphoma. Type I IFN gene expression has been reported to correlate between peripheral blood and salivary gland tissue, whereas type II IFN gene expression in peripheral blood does not appear to reflect that in salivary gland tissue. SG, salivary gland; PB, peripheral blood. Created in Biorender. Ha, Y. (2026) https://BioRender.com/7s8aleu, accessed on 15 August 2026.

Among the IFN pathways involved in SjD, the type I IFN signature has been the most consistently characterized. Transcriptomic studies have reported that approximately 50–80% of patients with SjD exhibit interferon-stimulated gene (ISG) overexpression in blood or immune cell subsets, supporting the presence of a type I IFN-high molecular subset [12,15]. Circulating IFN-α activity is increased in a substantial subset of patients with SjD, and serum IFN-α2 levels correlate closely with blood ISG expression [16,17,18]. In addition, systemic type I IFN activation is associated with higher B-cell activating factor (BAFF) expression, more pronounced serological abnormalities, and higher clinical disease activity, further supporting the concept that systemic type I IFN activation is a major molecular feature in SjD (Figure 1) [19]. Type I IFN activity is not restricted to the systemic circulation. Local IFN pathway markers, such as MX1, RSAD2, and phosphorylated-STAT1, have been reported in salivary gland tissue and have even been proposed as adjunctive diagnostic markers in SjD [20,21,22]. These findings suggest that type I IFN activation in SjD occurs not only systemically but also within the target tissue microenvironment.

However, IFN dysregulation in SjD is not limited to type I pathways. Salivary gland tissue contains both type I- and type II-related ISGs [23,24,25]. Several studies suggest that local glandular inflammation is influenced by multiple IFN programs rather than a single uniform pathway [24,26]. Notably, type II predominant IFN activity is associated with higher focus scores in salivary gland tissues, and an elevated IFN-γ/IFN-α mRNA ratio has been linked to the risk of lymphoma development in SjD [25,26]. In peripheral blood, upregulation of type II IFN-associated transcriptional modules has not been observed in isolation but consistently co-occurs with type I IFN activation [15]. Together, these observations suggest that, compared with the peripheral blood, the salivary gland microenvironment may be relatively enriched for type II IFN biology. This compartmentalized interpretation of IFN biology in SjD is summarized in Figure 1.

Type III IFN represents an important, but relatively underexplored, component of the IFN network in patients with SjD because its biology is particularly relevant to epithelial barrier tissues. Type III IFNs are induced through overlapping IRF3/IRF7-dependent nucleic acid-sensing pathways, and they can amplify local antiviral-like programs without fully mirroring the biology of systemic type I IFN in epithelial tissues. Several studies have suggested that SGECs express receptors for type III IFNs and that type III IFNs are increased in minor salivary gland (MSG) tissues of patients with SjD [27,28]. Type III IFN can be induced by activating TLR3 and can increase the expression of CXCL10 and BAFF in SGECs, acting synergistically with IFN-α and thus supporting a potential epithelial amplification loop [28]. The relative contribution of each IFN class to the pathogenesis of SjD and which IFN pathway represents the optimal therapeutic target remains unresolved.

### 3.3. Cellular and Tissue Context of IFN Activation

The compartmental differences outlined above are likely to reflect differences in the dominant cellular sources, responding cell populations, and the local microenvironmental context of IFN activation in SjD. In particular, SGECs are increasingly regarded not merely as passive targets of immune-mediated injury, but as active participants in the initiation and propagation of the disease [13,29]. In response to endogenous or exogenous danger signals, SGECs can engage TLR-dependent and -independent pathways, acquire antigen-presenting cell (APC)-like features, and produce a proinflammatory environment that includes interleukin (IL)-6, BAFF, CXCL12, and type I and III IFNs. In addition, epithelial apoptosis can release autoantigens and apoptotic particles, further promoting local immune activation.

At the level of immune cells, plasmacytoid dendritic cells (pDCs), conventional dendritic cells (DCs), macrophages, monocytes, T cells, and B cells contribute to the inflammatory network associated with IFNs in SjD [13]. As major producers of type I IFNs, pDCs can be redistributed from the peripheral blood to inflamed glandular tissues [30], where they can be activated by nucleic acid-containing ligands and inflammatory cues derived from SGECs. Type I IFNs, in turn, amplify local immune responses by promoting antigen presentation, activating DCs, and supporting differentiation of effector T-cell subsets and T follicular helper (Tfh) cells [13,31], thus facilitating B-cell activation and autoantibody production. Immune complexes formed by autoantibodies and released autoantigens can further stimulate pDCs, creating a self-perpetuating inflammatory loop.

Recent studies also suggest that type I IFN-induced ectopic expression of lysosome-associated membrane protein 3 (LAMP3) in SGECs may enhance TLR7 expression [32], whereas impaired METTL3-mediated m^6^A modification may promote double-stranded RNA accumulation [33], thus contributing to the amplification of IFN signaling within the glandular microenvironment. This concept is consistent with the view of SjD as an autoimmune epithelitis, in which epithelial stress and immune activation are closely coupled [34].

Type II IFN signaling adds another layer of tissue-specific immune activation [25]. T cells and NK cells are considered the main sources of IFN-γ in SjD [35,36], and recent evidence suggests that CD8^+^ tissue-resident memory T cells expanded in salivary gland tissue may directly contribute to glandular injury by secreting granzyme and IFN-γ [37]. Similar IFN-γ-driven epithelial damage has also been described in other target tissues, such as the conjunctiva [38]. Overall, IFN activation in SjD spans both salivary gland tissue and peripheral blood; however, blood-based IFN signatures primarily reflect systemic type I IFN activity, whereas glandular IFN signatures more directly capture integrated local IFN activity associated with exocrine glandular epithelial stress, infiltrating immune cells, and local inflammatory injury.

### 3.4. Clinical Relevance of IFN Signatures in SjD

Clinically, IFN signatures help define biologically distinct subgroups of SjD. Patients with higher systemic IFN activation more frequently exhibit anti-Ro positivity, hypergammaglobulinemia, upregulation of BAFF, extra-glandular manifestations, high EULAR Sjögren’s Syndrome Disease Activity Index (ESSDAI) scores, and high risk of lymphoma [16,19,39]. Recent cluster analysis suggests that a high IFN signature may identify patients with a B-cell-active phenotype who are at increased risk of systemic progression and the need for immunosuppressive treatment [40]. However, subjective symptom burden, including sicca, limb pain, or fatigue, does not necessarily parallel IFN activity. These clinical and immunological differences support the concept of IFN-based endotype stratification in SjD, as summarized in Figure 1.

Local tissue IFN activation can provide additional prognostic information. In salivary gland tissue, markers such as an elevated IFN-γ/IFN-α ratio and increased expression of ISG15 have also been associated with SjD-related lymphoma, supporting the potential value of IFN-related profiling for risk stratification [25,41]. In addition, IFN-driven immune activation may have additional clinical implications, as type I IFN-high states have been linked to trained immunity phenotypes in monocytes, which could contribute to the increased cardiovascular risk observed in SjD [42]. IFN-γ-inducible metabolites, including kynurenines and neopterin, have been associated with disease activity, glandular dysfunction, autoantibody positivity, and inflammatory burden [43]. Together, these findings suggest that IFN-related biomarkers can contribute to prognostic stratification, particularly in patients with biologically active or lymphoma-prone disease.

IFN signatures are also closely related to SjD endotypes at the tissue level. mRNA-based studies have suggested compartmental differences, with type I IFN signatures predominating in peripheral blood and type II IFN-related signatures relatively enriched in MSGs [25]. Protein-based clustering analyses have identified heterogeneous glandular IFN patterns, including type I-predominant, type II-predominant, and mixed IFN patterns [26]. Additionally, recent single-cell and spatial transcriptomic analyses have further indicated autoantibody-associated variation in glandular IFN activation, with stronger type I and type II IFN signatures in anti-Ro-positive subtypes than in anti-centromere-positive disease [44].

## 4. mtRNA Biology and Immunostimulatory Properties

Unless otherwise stated, the mechanistic evidence summarized in this section comes mainly from basic experimental studies and non-SjD disease contexts and is presented as a general model for understanding mtRNA dysregulation in SjD.

### 4.1. Biogenesis and Processing of mtRNAs

Mitochondria are semiautonomous, double-membrane organelles that play an essential role in energy metabolism, redox regulation, apoptosis, calcium homeostasis, and innate immune signaling [45]. In addition to these canonical functions, mitochondria are increasingly recognized as important hubs in innate immunity because mitochondria-derived molecules can act as endogenous danger signals under conditions of cellular stress or injury.

**Figure 2:**
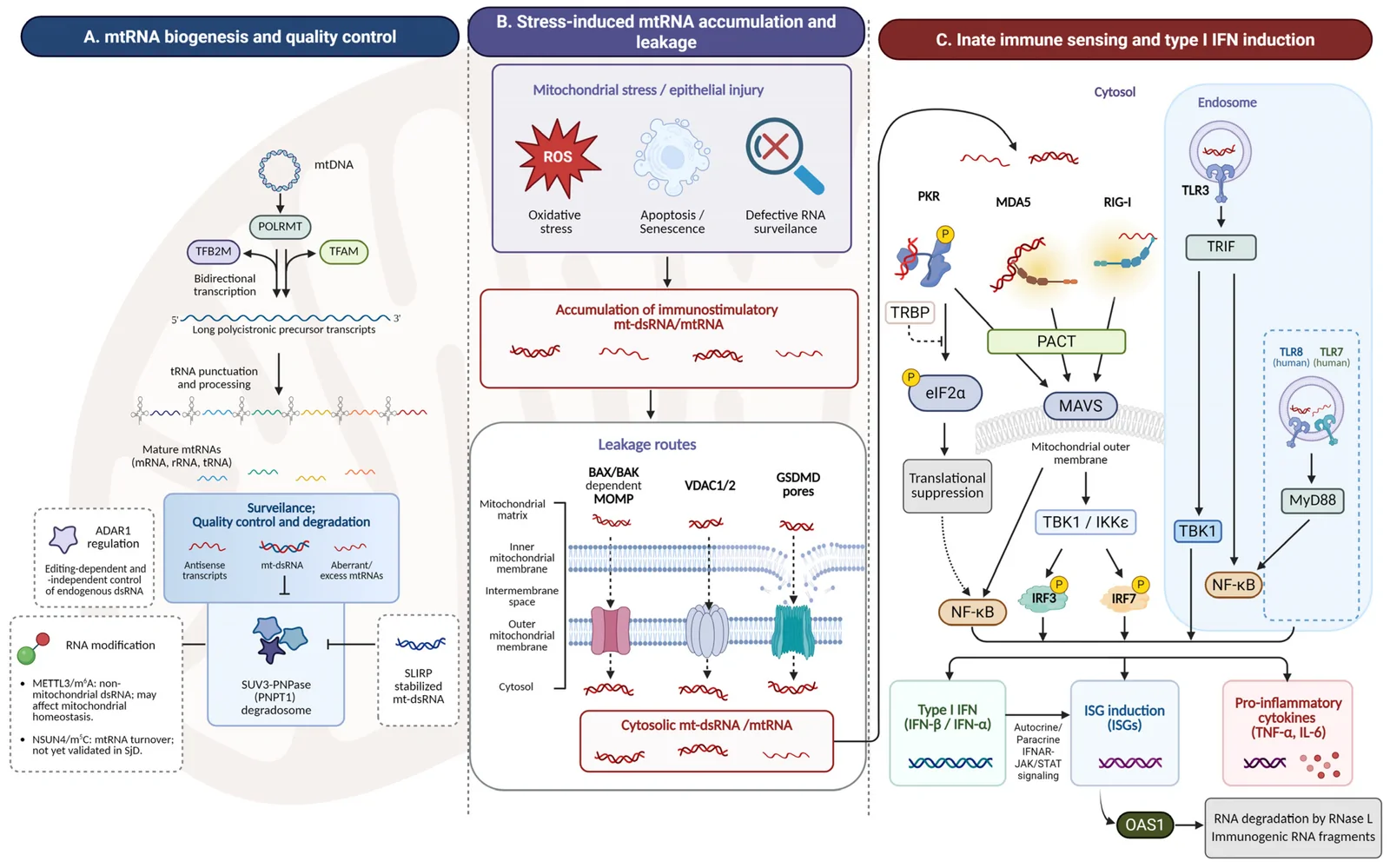
mtRNA biogenesis, cytosolic leakage, and innate immune sensing leading to type I IFN production. (**A**) Bidirectional transcription of mtDNA generates mtRNAs, which subsequently undergo processing, modification, and quality control. Disruption of RNA processing, surveillance, or degradation can lead to the accumulation of aberrant mtRNA species, including mt-dsRNA. (**B**) Mitochondrial or epithelial stress may further increase the accumulation of immunostimulatory mtRNA and facilitate its cytosolic release through distinct mitochondrial membrane-permeabilization mechanisms, including BAX/BAK-dependent mitochondrial outer membrane permeabilization, VDAC-dependent membrane permeabilization, and pore formation by activated GSDMD. (**C**) Cytosolic mtRNAs can activate PKR or engage MDA5 and RIG-I, which signal through MAVS, whereas endosomal mtRNAs may engage TLR3, TLR7, or TLR8. Collectively, these pathways promote type I IFN production, ISG induction, and proinflammatory cytokine production. Solid arrows indicate signaling or regulatory relationships, whereas dashed blunt-ended lines indicate inhibitory effects. Colors are used for visual distinction only. BAK, BCL-2 antagonist/killer 1; BAX, BCL-2-associated X protein; GSDMD, gasdermin D; IFN, interferon; ISG, interferon-stimulated gene; MAVS, mitochondrial antiviral-signaling protein; MDA5, melanoma differentiation-associated protein 5; mtDNA, mitochondrial DNA; mt-dsRNA, mitochondrial double-stranded RNA; mtRNA, mitochondrial RNA; NF-κB, nuclear factor-kappa B; OAS1, 2′-5′-oligoadenylate synthetase 1; PACT, protein activator of PKR; PKR, protein kinase R; POLRMT, mitochondrial RNA polymerase; RIG-I, retinoic acid-inducible gene I; SjD, Sjögren’s disease; TFAM, mitochondrial transcription factor A; TFB2M, mitochondrial transcription factor B2; TLR, Toll-like receptor; TRBP, TAR RNA-binding protein; VDAC, voltage-dependent anion channel. Created in Biorender. Ha, Y. (2026) https://BioRender.com/o8gf75f, accessed on 15 August 2026.

The mitochondrial genome is a compact circular DNA molecule organized into heavy and light strands and lacks introns [46]. Mitochondrial transcription is mediated by mitochondrial RNA polymerase (POLRMT) in cooperation with mitochondrial transcription factor A (TFAM) and mitochondrial transcription factor B2 (TFB2M) (Figure 2A). mtRNAs are transcribed bidirectionally, almost entirely into long polycistronic precursor RNAs, which are later processed into mature RNAs. Under physiological conditions, the abundance of mtRNAs and immunogenicity are constrained by coordinated post-transcriptional processing, maturation, and quality-control systems. Polycistronic precursors of mtRNAs are processed through the tRNA punctuation mechanism and undergo further maturation, whereas aberrant or excess mtRNA species, including antisense transcripts and mt-dsRNAs, are restricted by the mtRNA surveillance and degradation pathways, particularly the SUV3–PNPase (PNPT1) degradosome axis [47,48]. In addition to degradosome-mediated surveillance, mt-dsRNA homeostasis is influenced by broader endogenous dsRNA-tolerance mechanisms and mtRNA-specific RNA modification pathways. ADAR1 limits self-dsRNA immunogenicity and has recently been shown to regulate both nuclear and mitochondrial dsRNA formation through editing-dependent and editing-independent mechanisms, despite limited A-to-I editing of mitochondria-derived RNAs [49,50]. More recently, NSUN4-mediated m^5^C modification of mtRNAs has been shown to promote C1QBP/PNPT1-dependent mtRNA turnover, thus limiting mt-dsRNA accumulation, cytosolic release, and innate immune activation [51]. However, direct involvement in SjD-associated mtRNA dysregulation remains to be defined. In parallel, RNA-stabilizing factors such as SLIRP may promote the persistence of mt-dsRNAs and amplify IFN signaling through a positive-feedback mechanism [52]. Disruption of these processes can promote the accumulation of immunostimulatory RNA species, providing a basis for the inflammatory properties of mtRNAs discussed in the following sections (Table 2 and Table S1).

### 4.2. Formation, Cytosolic Leakage, and Extracellular Release of Mitochondrial Double-Stranded RNAs

Because the heavy and light strands generate long complementary RNA species through bidirectional transcription, these transcripts can hybridize to form mt-dsRNAs [53]. Under physiological conditions, surveillance systems rapidly limit the accumulation of these duplex RNAs. However, impairment of this machinery can lead to a marked accumulation of mt-dsRNA [48]. Deficiency of SUV3 or PNPase (PNPT1), the core components of the mitochondrial degradosome, leads to marked accumulation of dsRNA, and perturbation of PNPase-dependent control has been associated with cytosolic leakage of mt-dsRNA [54].

mtRNAs are normally confined within the mitochondria and are isolated from cytosolic nucleic acid sensors. However, cytosolic escape of mtRNAs can occur under conditions of mitochondrial stress, apoptosis, senescence, or defects in RNA quality-control pathways (Figure 2B) [55,56]. One proposed route involves BAX/BAK-dependent mitochondrial outer membrane permeabilization (MOMP), which allows inner membrane herniation and exposure of matrix contents to the cytosol, including mtRNAs [55,57]. VDAC1/2 and PHB1/2 have also been implicated in regulating the release of mitochondrial nucleic acids to the cytosol [58]. In addition, gasdermin D (GSDMD)-mediated pore formation has been proposed as an additional route for the release of cytoplasmic mtRNA [59].

Cytosolic mt-dsRNAs are thus recognized as danger-associated signals accessible to intracellular innate immune sensors [55,60]. Cytosolic accumulation of mt-dsRNA is not restricted to a single disease model but can occur under various stress conditions and can contribute to inflammatory phenotypes, including senescence-associated secretory phenotype [54,55,61,62].

mtRNAs can also be released into the extracellular space through cell injury or via export associated with extracellular vesicles (EVs) [53]. Due to their relative structural stability, extracellular mt-dsRNAs can retain immunostimulatory potential and promote inflammatory signaling in recipient cells [53,63,64]. Together, these pathways provide a mechanistic basis through which normally compartmentalized mtRNAs become accessible to innate immune sensors of the cytosol or endoplasm. Therefore, they may function not only as biomarkers of mitochondrial stress but also as mediators of paracrine immune activation.

### 4.3. Recognition of mtRNAs by Innate Immune Sensors and Their Role as Endogenous Triggers of Inflammation

When mtRNAs, particularly mt-dsRNAs, become mislocalized beyond mitochondrial confinement, they can be recognized as virus-mimicking ligands by innate RNA sensors (Figure 2C). Cytosolic sensors relevant to mtRNA sensing include MDA5, RIG-I, and protein kinase R (PKR), whereas endosomal RNA sensors include TLR3 and TLR7 [47,58,59,60]. In humans, mtRNAs containing UR/URR motifs can also activate TLR8-dependent signaling [65]. The structural features of cytosolic mtRNAs are important to determine which sensors are engaged. Because mt-dsRNAs can form long complementary duplexes, they are most plausibly linked to sensors that preferentially recognize extended dsRNA structures. In this regard, cytosolic mt-dsRNAs are primarily associated with PKR- and MDA5-dependent sensing, although RIG-I may also contribute in specific contexts [46,66].

The downstream consequences of mtRNA sensing vary by sensor type and cellular compartment. Recognition of mtRNAs by MDA5 or RIG-I induces conformational changes that expose their caspase recruitment domains (CARDs), enabling interaction with mitochondrial antiviral signaling protein (MAVS) on the outer mitochondrial membrane and triggering downstream TBK1/IKKε-, IRF3/IRF7-, and NF-κB-dependent signaling [53]. These pathways ultimately promote the expression of type I IFNs, ISGs, and proinflammatory mediators such as TNF-α and IL-6. In parallel, cytosolic mt-dsRNAs can also bind to PKR, leading to its autophosphorylation and downstream phosphorylation of eIF2α, which suppresses global protein synthesis while further amplifying NF-κB-mediated inflammatory programs [55,67].

As listed in Table 2 and Table S1, a variety of proteins involved in mtRNA biogenesis and processing, mitochondrial membrane transport, dsRNA sensors, and downstream or modulatory factors can shape IFN amplification. These include OAS1, PACT, TRBP, and PARP9 (Figure 2). Although the strength of SjD-specific evidence varies across these molecules, together they illustrate the mechanistic extent of the mtRNA/type I IFN axis beyond a single sensor pathway and provide a mechanistic framework for understanding how mtRNA dysregulation may drive chronic IFN activation in SjD.

These mechanisms primarily explain how mtRNA is detected after reaching the cytosol or endosomes within the cell in which it accumulates. By contrast, extracellular mtRNA would need to be internalized before engaging intracellular RNA sensors. Evidence from extracellular dsRNA models indicates that receptor-mediated endocytosis and EV uptake are plausible entry routes, although direct uptake mechanisms for free extracellular mtRNA have not been established in SjD.

More specifically, free extracellular dsRNA may be internalized through scavenger receptor-mediated endocytosis; in human monocytes, scavenger receptor expressed by endothelial cells I (SREC-I) facilitates the delivery of extracellular dsRNA to TLR3-containing endosomes [68]. Following endocytic uptake, dsRNA retained within endosomal compartments may activate TLR3 [68]. Single-stranded mtRNA species may also engage endosomal TLR7 or TLR8, although how extracellular mtRNA reaches these receptors remains incompletely understood [65,69]. Cytosolic recognition requires an additional transport step across the endolysosomal membrane. SIDT2 has been shown to transfer internalized extracellular dsRNA from endolysosomal compartments into the cytosol, thereby enabling RIG-I-like receptor signaling [70]. For EV-associated RNA, endosomal escape of nucleic acid cargo has also been demonstrated in engineered EV systems [71]; however, whether native mtRNA-containing EVs deliver their cargo to cytosolic MDA5, RIG-I, or PKR remains unresolved. In a non-SjD model, hepatocyte-derived exosomal mt-dsRNA activated TLR3 in Kupffer cells, providing direct evidence that EV-associated mtRNA can transmit inflammatory signals between cell types [72].

The recipient cell populations and sensing routes have not been established directly in SjD. SGECs are plausible local recipients because they possess functional TLR3- and TLR7-related RNA-sensing pathways and respond strongly to dsRNA stimulation [3,32,73,74,75]. Among immune cells, macrophages have the strongest supporting evidence from non-SjD models: exosomal mt-dsRNA activates TLR3 in Kupffer cells, whereas endogenous mtRNA can promote TLR7-dependent IFN production in macrophages [69,72]. Conventional DC subsets may detect dsRNA through TLR3, whereas pDCs and B cells may respond to single-stranded or fragmented RNA through endosomal TLR7 [30,76,77]. Although these cell populations are implicated in the SjD inflammatory microenvironment, direct uptake and sensing of extracellular mtRNA have not been demonstrated. Thus, whether extracellular mtRNA mediates epithelial-to-epithelial signaling, epithelial-to-myeloid signaling, or broader communication involving adaptive immune cells remains an important question for future investigation.

**Table 2:** Sjögren’s disease-related regulators and sensors of the mtRNA-interferon axis.

Category	Protein	Evidence	References
mtRNA generation and processing	POLRMT	Mitochondrial transcription; pharmacological inhibition may reduce mt-dsRNA burden in SGECs.	[3]
	SUV3	Mitochondrial RNA helicase that limits mt-dsRNA accumulation; reduced SUV3 in juvenile SjD is associated with matrix accumulation of mtRNA and IFN activation.	[46,48,58,78]
Endogenous dsRNA modification and regulation	ADAR1	ADAR1 limits endogenous dsRNA accumulation and self-RNA immunogenicity. Altered RNA-editing profiles have been reported in SjD. ADAR1 can regulate both nuclear and mitochondrial dsRNAs formation through editing-dependent and editing-independent mechanisms.	[49,50]
	METTL3	m^6^A modification restrains endogenous cytoplasmic dsRNA accumulation and sustained type I IFN induction in SGECs, although this mechanism is not mtRNA-specific. METTL3/m^6^A may indirectly influence mitochondrial homeostasis.	[33,79]
mtRNA stabilization factors	SLIRP	Stabilizes mt-dsRNA and amplifies MDA5-dependent antiviral/IFN signaling through a positive-feedback loop. SLIRP is upregulated in CD14^+^ monocytes from SjD.	[52]
Gasdermin-mediated pore formation	GSDMD	Increased in SjD salivary glands and SGECs; type I IFN promotes GSDMD-associated pyroptosis. GSDMD-associated pore formation may permit mitochondrial DAMP or mtRNA release.	[59,80,81]
Cytosolic RNA sensing	MDA5 (*IFIH1*)	Senses long cytosolic dsRNA and activates MAVS-dependent IFN signaling; upregulated in pDCs and monocytes from IFN-positive SjD.	[52,76]
	RIG-I (*DDX58*)	Preferentially senses 5′-triphosphate/diphosphate-containing or blunt-ended structured RNA and activates MAVS-dependent IFN signaling; upregulated in pDCs and monocytes from IFN-positive SjD. RIG-I-related endogenous RNA-sensing pathway signatures are enriched in SjD salivary glands linked to RF and anti-La/SSB seropositivity.	[76,82]
	PKR (*EIF2AK2*)	Binds endogenous mt-dsRNA and activates eIF2α stress signaling; linked to mt-dsRNA/p-PKR/ISG activation and epithelial dysfunction in SjD-related salivary gland models/tissues.	[66,83,84]
dsRNA-responsive effector	OAS1	dsRNA-activated enzyme that synthesizes 2′-5′ oligoadenylates, activating RNase L, which degrades cellular RNA and amplifies IFN responses; a genetic susceptibility locus for SjD; consistently upregulated component of published IFN-signature and biomarker panels.	[85,86,87]
RNA-sensing cofactors and RLR modulators	PACT (*PRKRA*)	A dsRNA-binding cofactor of RIG-I and MDA5, stimulating RIG-I ATPase activity and promoting MDA5 oligomerization; can restrain aberrant PKR activation by endogenous dsRNA; cytoplasmic PKR–PACT colocalization is reported in SjD salivary glands.	[84,88,89,90]
	TRBP (*TARBP2*)	A dsRNA-binding PKR inhibitor/modulator that negatively regulates RLR signaling, partly through inhibition of PKR-dependent antiviral stress granule formation; targets MAVS to disrupt RIG-I–MAVS and MAVS–TRAF3 associations; nuclear localization of TRBP is reported in SjD salivary glands.	[84]
Endosomal RNA sensor	TLR3	Endosomal dsRNA/endogenous RNA sensor; TLR3 is functionally active in SjD salivary glands/SGECs; poly(I:C)-TLR3 stimulation induces type I and III IFNs, autoantigen redistribution, glandular dysfunction and SGEC anoikis; exosomal mt-dsRNA can activate TLR3 and TLR3-linked mitochondrial dysfunction.	[28,72,73,74,75,91]
	TLR7	Endosomal ssRNA sensor; increased in IFN-positive SjD pDCs/monocytes and in SjD salivary glands, where it amplifies type I IFN production. mtRNA can drive interferon production through TLR7.	[32,69,76,92,93]
IFN-amplifying regulator	PARP9	A non-canonical RNA sensor amplifying type I IFN production through the PI3K/AKT3-IRF3/IRF7 pathway; overexpressed in salivary glands and hypomethylated/upregulated in CD19^+^ B cells of SjD.	[94,95,96]

SGEC, salivary gland epithelial cell; SjD, Sjögren’s disease; IFN, interferon; DAMP, damage-associated molecular pattern; pDC, plasmacytoid dendritic cell; RF, rheumatoid factor; RLR, RIG-I-like receptor.

## 5. The mtRNA/Type I IFN Axis in SjD: Cellular and Molecular Mechanisms

### 5.1. Epithelial Stress and Mitochondrial Dysfunction in SjD

In SjD, exocrine glandular dysfunction reflects a combination of acinar and ductal epithelial activation, apoptosis, senescence, altered progenitor homeostasis, and dysregulation of innate immune pathways such as NF-κB, inflammasome, and IFN signaling [13,97]. Transcriptomic and ultrastructural studies suggest altered mitochondrial dynamics and metabolism in salivary gland tissue from patients with SjD, including altered mitochondria-related gene expression and structural abnormalities such as swollen mitochondria, cristae disruption, and autophagosome accumulation [98,99].

Oxidative stress is likely to be a key intermediate in these processes. SjD has been reported to be associated with increased markers of oxidative stress, and deletion of the mitochondrial antioxidant enzyme SOD2 in salivary gland ductal cells induces sicca-like glandular hypofunction, even in the absence of an autoimmune background [100,101]. At the immune-cell level, recent data also suggest systemic mitochondrial dysfunction and altered mitophagic signatures in SjD, including associations with fatigue phenotypes [102].

Although these studies do not prove a direct association with mtRNA release, they establish a biologically plausible background in which mitochondrial nucleic acids may become increasingly available for innate immune sensing during states of epithelial and mitochondrial stress. IFNs may further aggravate this stressed state reciprocally, although direct evidence is limited in SjD. IFN-γ induces mitochondrial dysfunction and oxidative stress in myositis models, whereas type I IFNs potentiate mitochondrial stress in mycobacterial infection and aged mutator mouse models [103,104,105]. These observations collectively suggest that, in SjD, IFN signaling itself can perpetuate mitochondrial stress, thus sustaining a self-reinforcing pathological cycle.

### 5.2. Innate Immune Activation Mediated by mtRNA

In this stressed epithelial context, SGECs are chronically exposed to inflammatory and oxidative stress, which may lead to mitochondrial dysfunction and abnormal handling of mtRNA [3,99]. SjD-related studies suggest that accumulated or mislocalized mtRNAs can amplify innate immune responses within the glandular microenvironment (Table 2).

mt-dsRNA levels were shown to be elevated in the salivary glands of aged non-obese diabetic (NOD) mice with salivary dysfunction and in MSG biopsies of patients with SjD [3]. Elevated mt-dsRNA immunostaining was co-localized with p-PKR and ISG expression in ductal and acinar epithelial cells [3]. In three-dimensional cultures of immortalized salivary gland cells, exogenous stimulation of dsRNA increased mt-dsRNA levels, induced ISG expression, enhanced PKR phosphorylation, and promoted SjD-like glandular phenotypes. These responses were attenuated by pretreatment with a JAK1 inhibitor and by suppression of mt-dsRNA using the POLRMT inhibitor, 2-C′-methyladenosine (CM) [3]. Together, these observations suggest that mtRNAs act as upstream activators of epithelial innate immune signaling in SGECs in SjD.

In parallel, impaired METTL3-mediated m^6^A RNA methylation in SGECs of patients with high IFN signatures leads to excessive accumulation of cytosolic dsRNA and sustained type I IFN induction, indicating that epitranscriptomic dysregulation of non-mitochondrial endogenous dsRNA may modulate IFN amplification in SjD [33]. In addition, recent non-SjD studies have suggested that METTL3/m^6^A could influence mitochondrial homeostasis through redox-sensitive mitochondrial quality-control pathways [79]. TLR3 is also relevant in this context, as poly(I:C)-responsive TLR3 pathways are functionally active in the salivary glands of patients with SjD and SGECs and may be amplified in part through mtRNA-dependent mechanisms [3,73]. TLR3-mediated responses induce type I and type III IFN programs, glandular dysfunction, and epithelial anoikis [28,74,91]. Recent data also suggest that mtRNA-stabilizing factors may modulate this pathway. In particular, SLIRP has been reported to be upregulated together with mtRNA in monocytes from patients with SjD, and downregulation of SLIRP reduced selected ISG expression in SGECs from patients with SjD [52].

Interestingly, oxidative stress can increase the immunogenic potential of mtRNA itself. Oxidized mtRNAs markedly potentiate IFNβ- and TNFα-inducing activity, with greater enhancement than oxidized mtDNA in THP-1 cells [106]. Given the oxidant-rich salivary gland microenvironment of SjD, oxidized mtRNAs may therefore contribute to epithelial IFN responses, although direct validation in SjD tissues is still needed. Overall, current SjD data support mtRNA-driven models, although the relative contribution of mtRNA versus mtDNA remains elusive.

### 5.3. Amplification of IFN Responses and Glandular Injury in SjD

This section integrates direct evidence of mtRNA dysregulation from SjD patient samples and SjD models with broader evidence on IFN-mediated epithelial injury; the latter is presented as mechanistic context rather than as evidence of an established mtRNA-dependent pathway. The initiating event in the mtRNA–type I IFN circuit in SjD has not been established and is unlikely to be uniform across all patients with SjD. Potential triggers may include viral or viral-like dsRNA exposure, oxidative or mitochondrial stress, and impaired mtRNA surveillance, although none has been demonstrated to initiate the circuit in patients with SjD [3,98,99,100,101]. In SjD-related SGEC models, the viral dsRNA mimic poly(I:C) induces mt-dsRNA accumulation, cytosolic release, and downstream innate immune activation [3]. This supports the biological plausibility of dsRNA-induced initiation but does not establish viral infection as the initiating event in human SjD. In non-SjD systems, loss of PNPase or SUV3 causes marked mt-dsRNA accumulation and cytosolic escape, leading to MDA5-dependent type I IFN signaling [46]. Reduced SUV3 expression has also been reported in an IFN-enriched monocyte subset from patients with childhood SjD, although its role in initiating glandular disease remains unproven [47]. Once mtRNA accumulation and IFN signaling are established, reciprocal reinforcement among mitochondrial stress, mtRNA dysregulation, and IFN-responsive pathways may convert a transient insult into persistent inflammation. Consistent with this model, mt-dsRNAs are elevated in saliva, tears, and primary SGECs from patients with SjD, as well as in salivary glands from SjD-prone mice [3]. In the latter study, acetylcholine partially suppressed poly(I:C)-induced epithelial changes through an mtRNA-dependent mechanism, whereas immunoglobulin G (IgG) derived from patients with SjD attenuated this protective effect [3].

This self-reinforcing inflammatory circuit is likely to converge with other IFN-driven modes of epithelial injury in SjD. Type I IFN promotes caspase-1 activation and GSDMD-driven pyroptosis in SGECs, linking IFN-rich epithelial stress to a membrane-injury program that may facilitate the release of damage-associated molecular patterns [80,81] (Table 2). Type II IFN can also contribute to epithelial injury: CD4^+^ T-cell-derived IFN-γ induces SGEC ferroptosis via JAK/STAT1-dependent suppression of system Xc^−^ (a cystine/glutamate antiporter), accompanied by AQP5 downregulation [107,108]. IFN-γ also induces lacrimal and ocular surface epithelial changes relevant to SjD-associated dry eye, including enhanced antigen-presentation-related pathways and epithelial cytotoxicity [109,110].

Moreover, this circuit may be further reinforced by crosstalk between the IFN pathways. Type III IFNs, which are elevated in SjD MSG tissues, enhance the expression of CXCL10 and BAFF in SGECs and act synergistically with IFN-α [27,28]. More broadly, type I and type II IFNs appear to contribute at different spatial levels in SjD. Type I IFN acts both systemically and locally, whereas IFN-γ functions more prominently as a local tissue effector cytokine (Figure 1). Animal model data support different but overlapping roles for type I and type II IFNs in SjD-like disease. In B6.Aec1/Aec2 mice, *Ifnar1* deficiency reduced inflammatory cell infiltration in both salivary and lacrimal glands without significantly altering humoral immune responses [111]. Conversely, IFN-γ or IFN-γ receptor deficiency in NOD-related models reduces the early pathology of the salivary gland, indicating a more direct local pathogenic role in sialadenitis [112]. Although these studies do not directly establish the causality of mtRNA, they provide a pathobiological context in which the mtRNA/IFN axis may converge on epithelial injury, antigen release, and perpetuation of local inflammation.

### 5.4. Integration with Adaptive Immunity and Systemic Autoimmunity

Direct evidence linking mtRNA dysregulation to adaptive immune activation in SjD remains limited. Accordingly, this section synthesizes SjD-specific data on IFN-driven immune responses with broader mechanistic evidence to propose how the mtRNA–IFN axis may extend beyond epithelial innate immunity to adaptive and systemic autoimmune responses. pDCs are considered important players that link local nucleic acid stress to amplification of type I IFN in SjD. Systemic type I IFN bioactivity in SjD has been associated with activation of pDCs [77], and pDCs can be recruited to target organs in patients with SjD [30]. Additionally, TLR7-related pathways are increased in IFN-positive circulating pDCs/monocytes and in salivary gland tissues of patients with SjD [32,76,92]. In inflamed glandular tissues, RNA-sensing pathways, including MDA5- and RIG-I-related programs, are enriched in IFN-positive SjD and can contribute to pDC-associated type I IFN amplification [86]. Although direct evidence for the role of mtRNAs in pDCs is still lacking, these findings support pDCs as an important type I IFN amplifier hub in SjD.

Type I IFN signaling also shapes T-cell responses relevant to humoral autoimmunity, promoting antigen presentation and supporting differentiation of effector T-cell subsets, including Tfh cells, thereby facilitating B-cell help and autoantibody production [31,113]. Transcriptional profiling of salivary gland CD4^+^ memory T cells has confirmed a germinal center Tfh signature in SjD [114]. Additionally, T-cell-derived IFN-γ contributes directly to SGEC injury in SjD [107,108]. Thus, IFN-rich glandular inflammation is likely to promote not only local epithelial activation but also T-cell-dependent immune responses.

B cells represent another major compartment in which IFN-driven amplification can be sustained. Salivary gland studies support a model in which the inflamed epithelium not only responds to IFN signaling but also provides chronic activation cues to infiltrating B cells within the glandular microenvironment [13]. In SGECs, IFN signaling induces BAFF expression [115], and SGECs from patients with SjD show an enhanced ability to support B-cell survival and activation, particularly after poly(I:C) stimulation [116]. Furthermore, PARP9, a non-canonical IFN-amplifying regulator (Table 2), is overexpressed in salivary glands and hypomethylated/upregulated in CD19^+^ B cells from patients with SjD, suggesting that IFN-associated amplification extends beyond epithelial cells to B-cell populations themselves [94,95,96].

In this framework, the mtRNA–IFN axis can provide a mechanistic bridge that links epithelial stress, activation of pDC, T-cell help, B-cell hyperactivity, and monocyte reprogramming with the systemic phenotype of SjD.

## 6. Translational and Clinical Evidence Supporting the mtRNA–Type I IFN Axis in SjD

### 6.1. Extracellular mtRNA Levels in Saliva and Plasma: Diagnostic and Clinical Correlates in SjD

**Figure 3:**
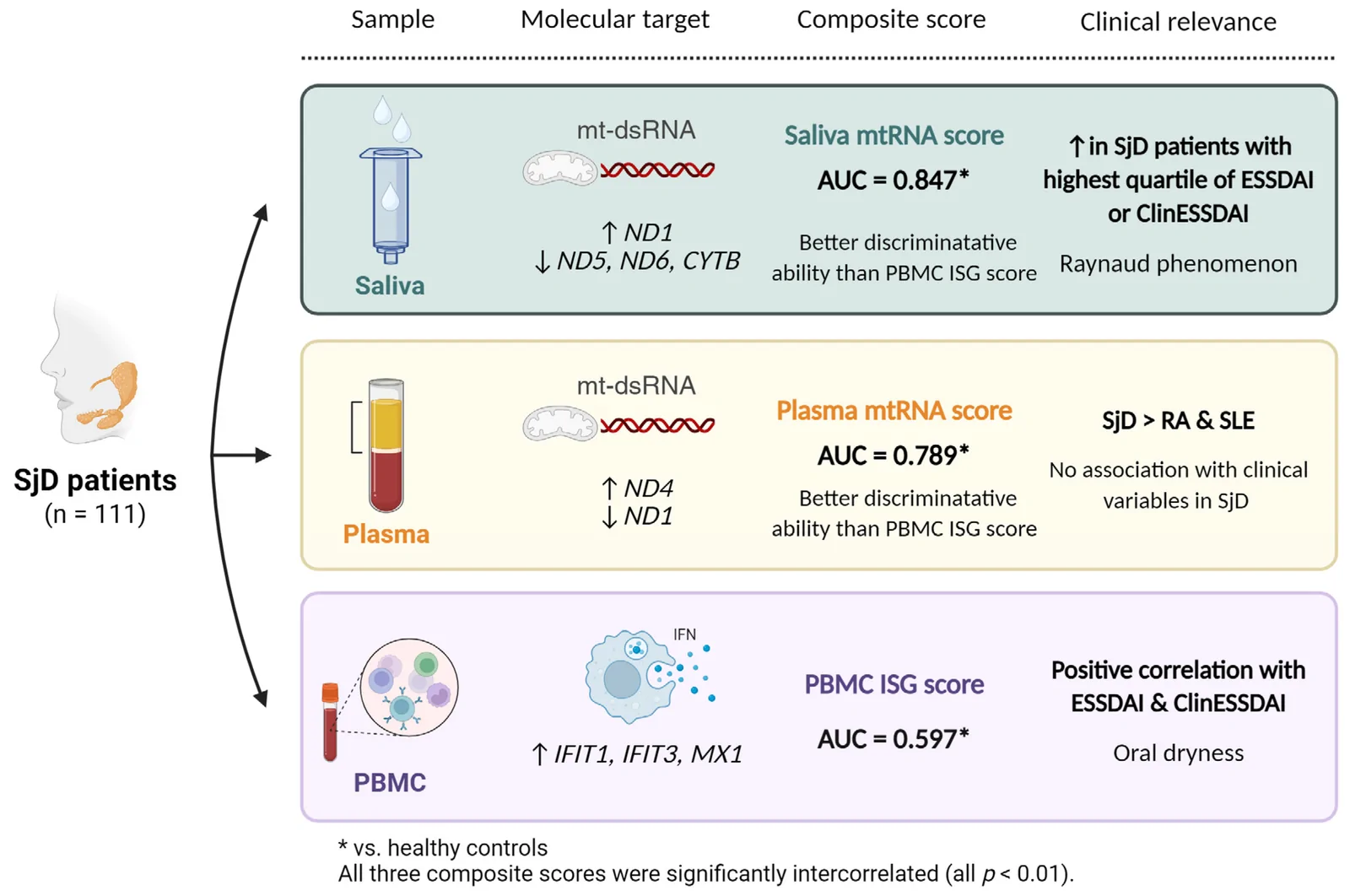
Salivary and plasma extracellular mtRNA in SjD. The figure summarizes mtRNA- and IFN-related findings in saliva, plasma, and PBMCs obtained from an overall cohort of 111 patients with SjD (saliva, *n* = 65; plasma, *n* = 79; PBMCs, *n* = 103). Salivary and plasma mtRNA yielded higher apparent AUCs than the PBMC ISG score, although the results were exploratory. Salivary mtRNA scores were higher in patients with the highest ESSDAI or ClinESSDAI quartile, whereas plasma mtRNA scores were higher in patients with SjD than in those with rheumatoid arthritis or systemic lupus erythematosus but were not associated with clinical variables within the SjD cohort. The salivary mtRNA, plasma mtRNA, and PBMC ISG composite scores were significantly intercorrelated. AUC values indicate discrimination between patients with SjD and healthy controls. Upward and downward arrows indicate increased and decreased expression levels, respectively; colors are used for visual distinction only. AUC, area under the receiver operating characteristic curve; ClinESSDAI, Clinical EULAR Sjögren’s Syndrome Disease Activity Index; ESSDAI, EULAR Sjögren’s Syndrome Disease Activity Index; IFN, interferon; ISG, interferon-stimulated gene; mt-dsRNA, mitochondrial double-stranded RNA; mtRNA, mitochondrial RNA; PBMC, peripheral blood mononuclear cell; SjD, Sjögren’s disease. Created in Biorender. Ha, Y. (2026) https://BioRender.com/ulnt4yf, accessed on 15 August 2026.

Evidence supporting extracellular mtRNA as a potential biomarker in SjD is currently based mainly on a single-center, cross-sectional study from our group [4]. The study included 111 patients with SjD and 35 healthy controls, although plasma, saliva, and peripheral blood mononuclear cell (PBMC) samples were available from only 79, 65, and 103 patients, respectively, and only 26 patients had all three biosamples. Composite salivary and plasma mtRNA scores showed higher apparent discrimination between patients with SjD and healthy controls than did the PBMC ISG score (Figure 3). However, because these scores were developed and evaluated in the same cohort without external validation, the findings should be considered exploratory, and their performance may have been overestimated.

Plasma mtRNA scores were also significantly higher in patients with SjD than those of patients with rheumatoid arthritis and systemic lupus erythematosus (SLE), with 40 patients included in each disease-control group. This difference remained significant after adjustment for immunoregulatory drug use. Nevertheless, medication patterns differed among the disease groups, and only 25 patients with SjD (22.5%) were receiving immunoregulatory drugs. Statistical adjustment may not fully account for differences in drug type, dose, duration, or treatment indication. Residual confounding by treatment therefore cannot be excluded, and the current findings do not establish the disease specificity of plasma mtRNA scores.

In the same study, salivary mtRNA, particularly salivary *ND1*, showed significant positive correlations with several PBMC ISGs, and the composite plasma mtRNA, saliva mtRNA, and PBMC ISG scores were significantly intercorrelated. Clinically, salivary mtRNA scores were associated with objective disease activity measures, including ESSDAI and clinical ESSDAI (ClinESSDAI). In addition, salivary mtRNA scores were significantly higher in patients in the highest quartile of ESSDAI or ClinESSDAI, whereas plasma mtRNA scores were not associated with clinical variables [4] (Figure 3). Altogether, these findings suggest, but do not establish, that salivary mtRNAs may reflect both gland-centered processes and systemic disease-related activity, whereas circulating mtRNAs may capture a broader systemic burden of mitochondrial nucleic acids. However, the cohort was recruited from a single Korean center, consisted predominantly of women, and generally had low systemic disease activity, limiting generalizability to men, other ethnic populations, and patients with more active disease. Overall, our study provides preliminary translational evidence for extracellular mtRNAs in patients with SjD, but independent validation in larger and more diverse prospective cohorts is required.

### 6.2. Remaining Gaps and Priorities for Validation

Several important gaps must be addressed before mtRNA-based biomarkers can be translated into clinical practice. First, further biological characterization is needed to determine the cellular origins, release pathways, and molecular forms of extracellular mtRNAs in saliva and plasma, including whether they differ among diseases with high IFN levels. Second, analytical validation should identify the most informative individual mtRNA species or composite scores and establish standardized pre-analytical handling, assay procedures, and reporting methods. Recent reviews of salivary biomarker research in SjD have also emphasized the importance of standardized pre-analytical protocols, harmonized assay procedures, and multicenter validation before any salivary biomarker platform is translated into clinical practice [117].

The clinical utility of mtRNA measurement also requires longitudinal validation. It remains unclear whether mtRNA levels precede disease flares, track progression over time, or predict response to IFN-targeting therapies. Consequently, salivary and plasma mtRNA measurements should be regarded as exploratory translational biomarkers rather than as ready-to-implement clinical biomarkers.

## 7. Therapeutic and Translational Implications

The proposed role of the mtRNA–type I IFN axis suggests that disease-relevant intervention may be possible at multiple levels, ranging from downstream IFN pathway blockade to upstream restoration of mtRNA homeostasis. However, the strength of evidence supporting these approaches currently varies markedly. IFNAR blockade has undergone randomized clinical evaluation in SjD (NCT05383677). JAK inhibition is supported by preclinical, ex vivo, and emerging human clinical evidence in SjD [118]. POLRMT inhibition is supported only by in vitro proof-of-concept evidence. Cholinergic modulation has limited mechanistic support in an SjD-like epithelial model, whereas mitochondrial quality-control approaches remain largely speculative. Endotype heterogeneity remains a major barrier in therapeutic development for patients with SjD. Nonetheless, extracellular mtRNA levels could provide translational value as biomarkers to identify biologically active type I IFN-associated subsets and to stratify patients who are more likely to benefit from pathway-directed therapies.

### 7.1. Targeting IFN Signaling and Downstream Effector Pathways: Emerging Clinical and Preclinical Evidence

Direct IFNAR1 blockade may attenuate IFN-driven autoimmune diseases [1]. Anifrolumab, an anti-IFNAR1 monoclonal antibody approved for SLE, was evaluated in the randomized, double-blind, placebo-controlled phase IIa trial involving 30 patients with SjD (ANISE-II; NCT05383677). Results presented at the 2026 EULAR Congress showed a higher Composite of Relevant Endpoints for Sjögren’s Syndrome (CRESS) response with anifrolumab than with placebo (80% versus 30%) [119]. However, the primary analysis was descriptive, and larger confirmatory trials and a full peer-reviewed publication are required. Additionally, whether mtRNA levels predict or mediate the therapeutic response to IFNAR blockade has not been established.

JAK inhibitors suppress both type I and type II IFN-driven responses through shared JAK1-containing signaling pathways. However, they act downstream of mtRNA sensing and do not directly inhibit mtRNA generation or accumulation. In SGEC models, JAK1 inhibition with upadacitinib attenuates poly(I:C)-induced mt-dsRNA accumulation, ISG induction, PKR activation, and loss of epithelial functional proteins [3]. In ex vivo studies using MSG tissue and primary SGECs, JAK inhibitors normalized aberrant pSTAT activation and exaggerated IFN-β responses [120]. A murine study of SjD also showed that tofacitinib improved salivary flow, reduced lymphocytic infiltration, and decreased effector T-cell subsets [121]. Human clinical evidence has also emerged. Liu et al. evaluated oral tofacitinib in a retrospective cohort of 112 patients and a prospective cohort of 10 patients with SjD [118]. Their ESSDAI scores significantly decreased in both cohorts. Nevertheless, the absence of randomized placebo controls, the small prospective cohort, and the potential influence of concomitant treatments preclude definitive conclusions regarding efficacy. JAK inhibitor use in SjD nonetheless warrants caution because SjD itself carries an increased risk of lymphoma, and the potential malignancy risk associated with JAK inhibitors has been debated.

TBK1-directed approaches have substantially less mature SjD-specific evidence. TBK1, a key node of the mtRNA–MAVS–IRF3 pathway, is upregulated in IFN-positive patients with SjD [122]. The TBK1 inhibitor amlexanox suppresses type I IFN production and B-cell differentiation in vitro, suggesting potential applicability in IFN-driven systemic autoimmune diseases including SjD [123], although systemic clinical evaluation in patients with SjD remains lacking.

### 7.2. Targeting mtRNA Generation and Homeostasis: In Vitro Proof-of-Concept Evidence

In SGEC models, 2-CM, a POLRMT inhibitor, reduced the burden of mt-dsRNA and ameliorated SjD-like epithelial phenotypes [3], providing proof-of-concept that pathogenic mtRNA accumulation can be targeted. However, because systemic POLRMT inhibition has safety concerns, the approach remains mechanistically informative. Experimental disruption of METTL3-mediated m^6^A methylation in SGECs of patients with SjD promotes excessive accumulation of cytoplasmic dsRNA and sustained induction of type I IFN, whereas m^6^A modification appears protective for salivary gland epithelial function in SjD [33]. Although epitranscriptomic modulation may be mechanistically attractive, therapeutic manipulation of endogenous dsRNA editing or degradation and direct blockade of dsRNA-sensing effectors remain experimental and require careful evaluation of safety and cell-type specificity.

### 7.3. Modulating Epithelial and Mitochondrial Stress Responses: Hypothesis-Generating Evidence

In SGEC models, acetylcholine partially suppressed poly(I:C)-induced mt-dsRNA accumulation and downstream epithelial changes, implicating muscarinic receptor signaling in the regulation of innate responses driven by mtRNA [3]. Muscarinic agonists such as pilocarpine and cevimeline are already being used clinically for sicca symptoms, although whether they attenuate mtRNA-mediated IFN activation in patients with SjD remains unknown. More broadly, vagus nerve stimulation has been reported to have potential benefit in SjD, but no evidence currently links this clinical effect to suppression of mtRNA or mtRNA-induced signaling in vivo [124].

In non-SjD cellular models, resveratrol may modulate mitochondrial stress and mt-dsRNA-associated immune activation by suppressing mt-dsRNA expression [125]. However, these experiments do not establish that resveratrol restores mitochondrial quality control or has an mtRNA-directed therapeutic effect in SjD. Additional evidence beyond SjD shows that mitophagy enhancement can suppress cytosolic mt-dsRNA leakage and downstream MAVS-associated inflammatory signaling [126]. These findings provide a biological rationale for further investigation but remain insufficient to support mitochondrial quality-control modulation as an mtRNA-directed therapeutic strategy in SjD.

## 8. Future Perspectives

Future research on the mtRNA–IFN axis in SjD should proceed along two complementary lines. Mechanistically, key priorities include defining the upstream drivers of pathological accumulation of mtRNAs in SGECs, such as oxidative stress, impaired RNA surveillance, and epitranscriptomic dysregulation, and evaluating additional candidate regulators of mt-dsRNA homeostasis, including PNPase, NSUN4, REXO2, VDAC1/2, BAX/BAK and FARSA, as summarized in Supplementary Table S1. It will also be important to determine whether oxidized mtRNAs enhance IFN-related responses in SjD tissues, as suggested by evidence from other disease contexts. The relative contributions of mtRNA versus mtDNA to IFN induction and the specific epithelial and immune cell subsets driving mtRNA sensing within the glandular microenvironment also warrant clarification through spatially resolved and single-cell approaches.

From a clinical perspective, longitudinal cohort studies are needed to determine whether mtRNA levels predict disease flare, therapeutic response, or lymphoma development, and whether salivary and plasma mtRNA measurements provide information beyond the established ISG-based measures. Standardization of sample collection, quantification methods, and a composite scoring system will be essential to ensure reproducibility across studies. Ultimately, integration of mtRNA data with transcriptomic, serological, and histopathological profiling may support molecular endotyping and more precise patient stratification in SjD.

## 9. Conclusions

Currently available evidence supports a mechanistic role for mtRNA dysregulation in SjD. Under epithelial stress, mt-dsRNAs can engage innate RNA-sensing pathways, amplify the type I IFN response, and contribute to glandular injury. Extracellular mtRNA levels in saliva and plasma correlate with IFN activity in PBMCs and objective disease measures, suggesting biomarker potential, although independent validation remains essential.

The mtRNA–type I IFN axis provides a biological basis for integrating epithelial stress and immune dysregulation, with implications for the development of clinical biomarker and targeted therapies in SjD. Realizing this potential will require mechanistic refinement, longitudinal validation, and methodological standardization.

## Figures and Tables

**Table 1:** Endogenous RNA candidates relevant to interferon activation in Sjögren’s disease: evidence basis, sensing pathways, and strength of SjD-specific evidence.

RNA Species	Evidence Basis: Direct vs. Extrapolated	Sensor/Pathway Engaged	Overall Strength of SjD-Specific Evidence *	References
Mitochondrial RNA (mtRNA)	Direct: mt-dsRNA is elevated in saliva and tears from SjD. dsRNA stress in salivary gland epithelial models increases mt-dsRNA/mtRNA and inflammatory readouts. Composite mtRNA scores in saliva and plasma discriminate SjD versus controls and correlate with PBMC IFN-stimulated gene (ISG) scores. Extrapolated: The involvement of additional mtRNA-sensing pathways is supported mainly by non-SjD experimental studies.	PKR–eIF2α/NF-κB signaling is supported in SjD-related models. MDA5/RIG-I–MAVS–type I IFN and endosomal TLR pathways are supported mainly by broader mechanistic evidence.	Moderate	[3,4]
Long interspersed nuclear element-1 (LINE-1)-derived RNA	Direct: Aberrant regulation of LINE-1 transcript in MSG of SjD across lymphoma risk strata. LINE-1-encoded open reading frame 2 protein (ORF2p) is detectable in MSG ductal epithelial cells with distribution varying by histopathologic severity. Extrapolated: A causal relationship between LINE-1 RNA and IFN activation in SjD has not been demonstrated.	No specific RNA sensor or causal IFN pathway has been established in SjD.	Low	[5,6]
Alu-derived short interspersed nuclear element (SINE) RNA	Direct: No direct functional evidence in SjD Extrapolated: Ro60 binds an RNA motif enriched in endogenous Alu. Increased Alu RNA levels are demonstrated in SLE patients with anti-Ro-positivity or high IFN signals. Alu RNA can trigger TLR7-dependent inflammatory cytokine production, including type I IFN.	TLR7-dependent inflammatory cytokine and type I IFN signaling, demonstrated outside SjD.	Very low	[7,8]
Endogenous retrovirus (ERV)-derived RNA	Direct: Increased transcripts from endogenous retrovirus (ERV) families, including ERVL, ERVK, and ERV1, in SjD saliva and MSG are reported. Extrapolated: Functional evidence linking these transcripts to IFN activation remains unavailable.	No RNA sensor or causal IFN pathway has been established in SjD.	Very low	[9]

SjD, Sjögren’s disease; IFN, interferon; MSG, minor salivary gland; SLE, systemic lupus erythematosus; PBMC, peripheral blood mononuclear cells. * Evidence levels were qualitatively defined as follows: moderate indicates replicated direct observations in SjD-derived samples or SjD-relevant models with partial mechanistic support; low indicates limited direct associations in SjD-derived samples without an established causal mechanism or sensing pathway; and very low indicates predominantly indirect or extrapolated evidence with minimal SjD-specific validation.

## Data Availability

No new data were created or analyzed in this study. Data sharing is not applicable to this article.

## Supplementary Materials

The following supporting information can be downloaded at: https://www.mdpi.com/article/10.3390/ijms27167295/s1. References [46,51,56,58,59,62,127,128,129,130,131,132,133] are cited in the supplementary materials.

## Abbreviations

The following abbreviations are used in this manuscript:

SjD	Sjögren’s disease
IFN	interferon
mtRNA	mitochondrial RNA
mt-dsRNA	mitochondrial double-stranded RNA
SGECs	salivary gland epithelial cells
MSG	minor salivary gland
ISG	interferon-stimulated gene
PBMC	peripheral blood mononuclear cell
JAK1	Janus kinase 1
TYK2	tyrosine kinase 2
STAT	signal transducer and activator of transcription
IRF9	interferon regulatory factor 9
ISGF3	interferon-stimulated gene factor 3 complex
ISRE	interferon-stimulated response element
GAS	γ-activated sequence
IFNLR1	interferon lambda receptor 1
IL10RB	interleukin-10 receptor subunit beta
IFNGR1	interferon gamma receptor 1
IFNGR2	interferon gamma receptor 2
TLR	Toll-like receptor
PRR	pattern-recognition receptor
RIG-I	retinoic acid-inducible gene I
MDA5	melanoma differentiation-associated gene 5
cGAS	cyclic GMP–AMP synthase
STING	stimulator of interferon genes
DC	dendritic cell
Tfh	T follicular helper
LAMP3	lysosome-associated membrane protein 3
METTL3	methyltransferase-like 3
m^6^A	N^6^-methyladenosine
SLE	systemic lupus erythematosus
BAFF	B-cell activating factor
ESSDAI	EULAR Sjögren’s syndrome Disease Activity Index
ClinESSDAI	clinical ESSDAI
SG	salivary gland
PB	peripheral blood
TFAM	mitochondrial transcription factor A
TFB2M	mitochondrial transcription factor B2
POLRMT	mitochondrial RNA polymerase
SUV3	mitochondrial RNA helicase SUV3
PNPase	polyribonucleotide nucleotidyltransferase
PNPT1	polyribonucleotide nucleotidyltransferase 1
ADAR1	adenosine deaminase acting on RNA 1
NSUN4	NOP2/Sun RNA methyltransferase 4
C1QBP	complement component 1 Q subcomponent binding protein
SLIRP	SRA stem-loop interacting RNA binding protein
BAX	BCL2-associated X protein
BAK	BCL2-antagonist/killer
MOMP	mitochondrial outer membrane permeabilization
VDAC1	voltage-dependent anion channel 1
VDAC2	voltage-dependent anion channel 2
PHB1	prohibitin 1
PHB2	prohibitin 2
GSDMD	gasdermin D
EV	extracellular vesicle
PKR	protein kinase R
CARD	caspase recruitment domains
MAVS	mitochondrial antiviral signaling protein
SREC-I	scavenger receptor expressed by endothelial cells I
TBK1	TANK-binding kinase 1
IKKε	I-kappa-B kinase epsilon
NF-κB	nuclear factor kappa-light-chain-enhancer of activated B cells
TNF-α	tumor necrosis factor alpha
IL-6	interleukin-6
OAS1	2′-5′-oligoadenylate synthetase 1
RNase L	ribonuclease L
PACT	protein activator of PKR
TRBP	TAR RNA-binding protein
PARP9	poly(ADP-ribose) polymerase family member 9
pDC	plasmacytoid dendritic cell
RF	rheumatoid factor
RLR	RIG-I-like receptor
DAMP	damage-associated molecular pattern
SOD2	superoxide dismutase 2
NOD	non-obese diabetic
AQP5	aquaporin-5
2-CM	2-C′-methyladenosine
