# Supplementary material for: Mitochondrial RNA–Type I Interferon Axis in Sjögren’s Disease: Molecular Mechanisms and Translational Implications
Source: Int J Mol Sci. 2026 Aug 15;27(16):7295. doi: 10.3390/ijms27167295 (PMC13512952; doi:10.3390/ijms27167295)
Supplement: Supplementary file 1 [file ijms-27-07295-s001.zip › ijms-4416306-supplementary.pdf]

Supplementary Table S1. Candidate regulators and sensors of the mtRNA-interferon axis supported by non-Sjögren disease mechanistic evidence

Category	Protein	Evidence	References
mtRNA processing and turnover	PNPase (PNPT1)	PNPase restricts mt-dsRNA accumulation and cytosolic escape. PNPase depletion causes cytosolic mt-dsRNA accumulation and MDA5–MAVS-dependent type I IFN induction.	[1-3]
mtRNA processing and turnover	NSUN4	NSUN4-mediated m^{5}C modification promotes C1QBP/PNPT1-dependent mtRNA turnover, thereby limiting mt-dsRNA accumulation, cytosolic release and innate immune activation.	[4]
mtRNA processing and turnover	REXO2	Mitochondrial oligoribonuclease that degrades short RNA generated by the SUV3–PNPase complex, preventing their cytosolic accumulation. A heterozygous dominant-negative REXO2 mutation (p.T132A) causes interferonopathy.	[5,6]
Inner mitochondrial membrane RNA transport	ANT2 (SLC25A5)	ANT2 functions as a mammalian mtRNA translocon and mediates mt-dsRNA efflux that can trigger innate immune activation.	[7]
Inner mitochondrial membrane proteins	PHB1/2	PHB1/2 inhibition attenuates the export of mt-dsRNAs to the cytosol.	[2]
Outer mitochondrial membrane permeabilisation	BAX and BAK	BAX/BAK-dependent mitochondrial permeabilisation is required for mtRNA release and IFN induction.	[1,2]
Outer mitochondrial membrane channel	VDAC1/2	VDAC1/2 are implicated in mt-dsRNA trafficking from mitochondria to cytosol when mitochondrial RNA quality control is perturbed.	[2]
Apoptosis-associated restraint of mtRNA signalling	Caspase-3/7	Suppress mtRNA-driven MDA5–MAVS–IRF3 type I IFN signalling during apoptosis.	[8]
Gasdermin-mediated pore formation	GSDME	GSDME forms mitochondrial membrane pores and promotes cytosolic mtRNA release in a non-SjD infection model.	[9]
RLR pathway regulator	LGP2 (DHX58)	Inhibits RIG-I-dependent responses and enhances MDA5-dependent responses through interaction with PACT; acts as a negative regulator of antiviral signalling by interfering with TRAF family protein.	[10,11]
Endogenous mt-dsRNA suppression	FARSA	The C. elegans orthologue FARS-1 and human FARSA have been implicated in downregulating endogenous dsRNAs, particularly mt-dsRNAs, together with RHA-2/DHX37. FARSA deficiency has also been associated with systemic inflammation, autoimmunity and increased IFN scores in children	[12,13]

IFN, interferon; miMOMP, minority mitochondrial outer membrane permeabilization; SjD, Sjögren disease; RLR, RIG-I-like receptor.

https://doi.org/10.3390/ijms27167295

References

1. Dhir, A.; Dhir, S.; Borowski, L.S.; Jimenez, L.; Teitell, M.; Rotig, A.; Crow, Y.J.; Rice, G.I.; Duffy, D.; Tamby, C. *et al.* Mitochondrial double-stranded RNA triggers antiviral signalling in humans. *Nature* **2018**, *560*, 238-242.
2. Krieger, M.R.; Abrahamian, M.; He, K.L.; Atamdede, S.; Hakimjavadi, H.; Momcilovic, M.; Ostrow, D.; Maggo, S.D.; Tsang, Y.P.; Gai, X. *et al.* Trafficking of mitochondrial double-stranded RNA from mitochondria to the cytosol. *Life Sci Alliance* **2024**, *7*.
3. Lopez-Polo, V.; Maus, M.; Zacharioudakis, E.; Lafarga, M.; Attolini, C.S.; Marques, F.D.M.; Kovatcheva, M.; Gavathiotis, E.; Serrano, M. Release of mitochondrial dsRNA into the cytosol is a key driver of the inflammatory phenotype of senescent cells. *Nat Commun* **2024**, *15*, 7378.
4. Kim, S.; Tan, S.; Ku, J.; Widowati, T.A.; Ku, D.; Lee, K.; You, K.; Kim, Y. RNA 5-methylcytosine marks mitochondrial double-stranded RNAs for degradation and cytosolic release. *Mol Cell* **2024**, *84*, 2935-2948 e2937.
5. Bruni, F.; Gramegna, P.; Oliveira, J.M.; Lightowlers, R.N.; Chrzanowska-Lightowlers, Z.M. REXO2 is an oligoribonuclease active in human mitochondria. *PLoS One* **2013**, *8*, e64670.
6. Idiiatullina, E.; Al-Azab, M.; Lin, M.; Hrovat-Schaale, K.; Liu, Z.; Li, X.; Guo, C.; Chen, X.; Li, Y.; Gao, S. *et al.* Heterozygous de novo dominant negative mutation of REXO2 results in interferonopathy. *Nat Commun* **2024**, *15*, 6685.
7. Wang, P.; Zhang, L.; Chen, S.; Li, R.; Liu, P.; Li, X.; Luo, H.; Huo, Y.; Zhang, Z.; Cai, Y. *et al.* ANT2 functions as a translocon for mitochondrial cross-membrane translocation of RNAs. *Cell Res* **2024**, *34*, 504-521.
8. Killarney, S.T.; Washart, R.; Soderquist, R.S.; Hoj, J.P.; Lebhar, J.; Lin, K.H.; Wood, K.C. Executioner caspases restrict mitochondrial RNA-driven Type I IFN induction during chemotherapy-induced apoptosis. *Nat Commun* **2023**, *14*, 1399.
9. Afaq, U.; Qudus, M.S.; Liu, S.; Wu, K.; Chen, Y.; Tian, M.; Wu, J. The role of gasdermin-mediated mitochondrial RNA release in amplifying secondary immune response during microbial infection. *Front Immunol* **2025**, *16*, 1668763.
10. Parisien, J.P.; Lenoir, J.J.; Mandhana, R.; Rodriguez, K.R.; Qian, K.; Bruns, A.M.; Horvath, C.M. RNA sensor LGP2 inhibits TRAF ubiquitin ligase to negatively regulate innate immune signaling. *EMBO Rep* **2018**, *19*.
11. Sanchez David, R.Y.; Combredet, C.; Najburg, V.; Millot, G.A.; Beauclair, G.; Schwikowski, B.; Leger, T.; Camadro, J.M.; Jacob, Y.; Bellalou, J. *et al.* LGP2 binds to PACT to regulate RIG-I- and MDA5-mediated antiviral responses. *Sci Signal* **2019**, *12*.
12. Sohn, J.; Jeon, M.; Lee, H.; Lim, J.; Ham, S.; Park, J.; Lee, J.; Jung, Y.; Lee, G.Y.; Min, H. *et al.* Phenylalanyl-tRNA synthetase FARS-1/FARSA balances longevity and immunity by downregulating endogenous mitochondrial double-stranded RNAs. *Mol Cell* **2026**,doi:10.1016/j.molcel.2026.04.030.
13. Charbit-Henrion, F.; Goguyer-Deschaumes, R.; Borensztajn, K.; Mirande, M.; Berthelet, J.; Rodrigues-Lima, F.; Khiat, A.; Fremond, M.L.; Bader-Meunier, B.; Rodari, M.M. *et al.* Systemic inflammatory syndrome in children with FARSA deficiency. *Clin Genet* **2022**, *101*, 552-558.

https://doi.org/10.3390/ijms27167295

Disclaimer/Publisher's Note: The statements, opinions and data contained in all publications are solely those of the individual author(s) and contributor(s) and not of MDPI and/or the editor(s). MDPI and/or the editor(s) disclaim responsibility for any injury to people or property resulting from any ideas, methods, instructions or products referred to in the content.

https://doi.org/10.3390/ijms27167295
